# CuO Nanowires Fabricated by Thermal Oxidation of Cu Foils towards Electrochemical Detection of Glucose

**DOI:** 10.3390/mi13112010

**Published:** 2022-11-18

**Authors:** Xun Cao

**Affiliations:** School of Materials Science and Engineering, Nanyang Technological University, 50 Nanyang Avenue, Singapore 639798, Singapore; CAOX0015@e.ntu.edu.sg

**Keywords:** copper oxide, thermal oxidation, non-enzymatic biosensor, glucose detection, metal/oxide composite

## Abstract

In view of the various stability issues and high cost of enzymatic glucose biosensors, non-enzymatic biosensors have received great attention in recent research and development. Copper oxide (CuO) nanowires (NWs) were fabricated on Cu foil substrate using a simple thermal oxidation method. The phase and morphology of the CuO NWs could be controlled by synthesis temperature. Variation in oxidation states enables CuO NWs to form Cu (III) species, which is crucial in catalysing the eletro-oxidation of glucose. The Cu-based metal/oxide composite electrode works as a non-enzymatic biosensor that adapts to the fast, dynamic change in glucose concentration, with a low saturation concentration (~0.7 mM) and a lower detection limit of 0.1 mM, making CuO NWs an excellent sensor towards impaired fasting glucose. The simplicity, cost-effectiveness and non-toxicity features of this study might make a way for potentially scalable application in glucose biosensing.

## 1. Introduction

Diabetes is a prevailing chronic disease around the world. According to the World Health Organization, ~422 million people are suffering from diabetes, which causes approximately 1.5 million deaths each year [1]. Diabetes mellitus (type 2) comprises 90% of diabetes cases resulting in heart disease, kidney failure and blindness [2,3,4,5,6]. Diabetic emergencies are prevented by routine measurement of physiological blood glucose levels several times a day, so the development of a highly sensitive, fast response, low cost, precise and reliable glucose biosensor will be of great help to clinical diagnosis.

Due to the intrinsic nature of proteins, enzyme-based glucose biosensors suffer from issues with losing activity and lacking of stability, which are constrained by many factors like pH, humidity, temperature and various detergents [7,8,9]. In addition, enzyme-based glucose sensors are costly. Therefore, the development of non-enzymatic glucose biosensors (NEGBs) has become a key research focus in recent years [10,11,12,13,14,15].

NEGBs are reported to feature fast and precise responses, excellent sensitivity and low cost [16], and are able to break through the limit on oxygen content in the environment [17]. Electrochemical NEGBs are based on a variety of materials to catalyze glucose oxidation reaction. The electrocatalysts can be classified into metals/alloys, oxides, hydroxides and carbon-based materials [18,19]. Compared with noble metals that can adopt the direct electrocatalytic oxidation of glucose, Cu-based materials become an active research area due to the advantages of low cost, non-toxicity and good electrical conductivity. In addition, Cu-based materials are shown to facilitate the inherent tendency for the oxidation of glucose. On the other hand, many oxide materials (especially transition metal oxides) show extraordinary performance in the electrocatalytic oxidation of glucose [20], owing to the high activity and adsorption capability contributed by varying oxidation states and formation of nanostructures.

In order to achieve both high-charge transfer rate and excellent electrocatalytic activity with reduced cost, numerous studies were carried out to fabricate metal/oxide composite sensors. Various types of surface nanostructures were achieved, such as nanoparticles, nanoflakes, nanowires (NWs), nanobelts, nanocubes, and nanoclusters [21]. These surface nanostructures generate large electrochemically active surface areas, which helps to increase the adsorption rate of glucose molecules onto the exposed sites. Table 1 lists various types of glucose biosensors with their performance.

In this work, Cu-based metal/oxide composite electrodes as NEGBs were fabricated via thermal oxidation. The biosensing mechanism is unveiled through eletrocatalytic reactions. The NWs synthesized at the optimum temperature has the maximum CuO/Cu_2_O composition ratio, which could respond quickly and reliably in low glucose concentrations (<1 mM). This simple and cheap method might make a way for potentially scalable application in glucose biosensing.

## 2. Materials and Methods

The chemicals used in this work include potassium hydroxide (KOH, 99%) flakes and D-(+)-glucose (C_6_H_12_O_6_, 99.5%) powders purchased from Sigma-Aldrich, which were separately dissolved in deionized water to form aqueous solutions of suitable concentrations for use in the electrochemical tests for glucose detection. The deionized water used was generated from Milli-Q system with ρ = 18.2 MΩ·cm.

### 2.1. Processing of Samples

High purity (99.9%) Cu foil tape was cut into pieces with a size of 20 × 5 mm^2^, and soaked in acetone for 8 h to remove organic contaminant on the Cu foils, followed by cleaning the Cu foils with ethanol for 30 min in an ultrasonic bath. Finally, the cleaned Cu foils were dried in an oven at 80 °C.

CuO NWs were fabricated by thermal oxidation of the Cu foils in a box furnace at atmospheric pressure, with a rising rate of 10 °C/min from room temperature to different target temperatures (400–700 °C) and kept isothermal for 2 h, followed by natural cooling to room temperature. In order to ensure both sides of the Cu foils were sufficiently reacted with equal amount of O_2_, the Cu foils were symmetrically folded and stood in the Al_2_O_3_ combustion boat. The final samples are denoted as CuO-400, CuO-500, CuO-550, CuO-600, CuO-650 and CuO-700 hereafter.

### 2.2. Characterisation and Testing Techniques

The crystalline phases of the samples were determined by X-ray diffraction (XRD) using a thin film X-ray diffractometer (XRD-6000, Shimadzu, Kyoto, Japan); the Cu Kα radiation was produced at 40 kV and 30 mA with λ ≈ 1.5405986 Å. All scans were performed within the range of 30° ≤ 2θ ≤ 85° under 2θ mode at an X-ray grazing angle of 1.0° with scanning rate of 0.5 °/min and a step size of 0.02°. The phase compositions were analyzed using Match! 3.12 software (Crystal Impact Inc., Bonn, Germany). The morphologies of the samples were characterized in a thermionic scanning electron microscope (SEM, JSM-6360A, JEOL, Tokyo, Japan), which was operated at an accelerating voltage of 20 kV under secondary electron imaging (SEI) mode with a working distance of 10 mm. The elemental compositions of the samples were confirmed by X-ray energy-dispersive spectroscopy (XEDS), which was performed at an accelerating voltage of 20 kV with an XEDS attachment (JED-2300, JEOL, Japan) on the SEM.

**Figure 1:**
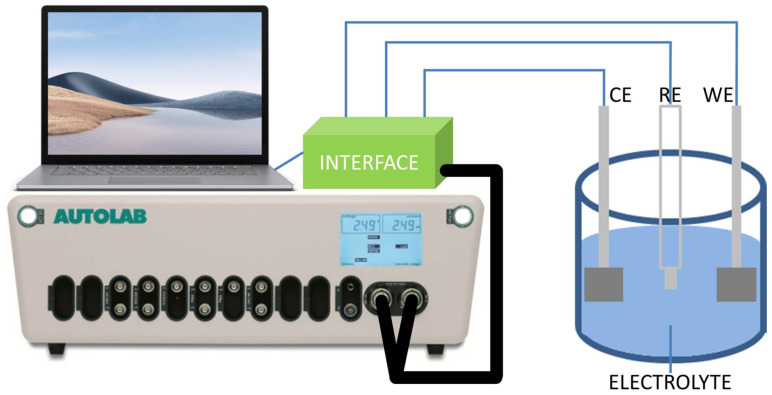
Schematic diagram of electrochemical testing setup.

The catalytic activities of the samples on glucose detection were tested using a three-electrode electrochemical workstation (PGSTAT302N, Metrohm Autolab, Utrecht, The Netherlands); the schematic diagram is illustrated in Figure 1. All electrochemical measurements were conducted under ambient conditions at room temperature and pressure. The as-fabricated samples, a Pt plate and an Ag/AgCl electrode were used as the working, counter and reference electrodes respectively. The electrolytes used were 0.1 M KOH with various concentrations of glucose. Cyclic voltammetry (CV) was conducted to study the electrocatalytic performance of the samples by measuring the respective oxidation current densities from the electrochemical reactions between the electrodes and the glucose solution at each specific potential. All tests were performed for 5 cycles within the potential range of −1 V to 1 V at the scan rate of 0.1 V/s with a step size of 2.44 mV, and only the third cycles were used for analysis. The chrono-amperometric (CA) response of the electrode to glucose was tested at the constant applied potential of 0.5 V in 0.1 M KOH solution under constant stirring. Suitable amounts of 0.1 mM glucose solution were intermittently injected at the rate of ~0.1 mM/min, so that the resultant concentrations of glucose varied from 0.1 mM to 10.0 mM.

## 3. Results and Discussions

### 3.1. Effects of Oxidation Temperatures on Phases and Morphologies

**Figure 2:**
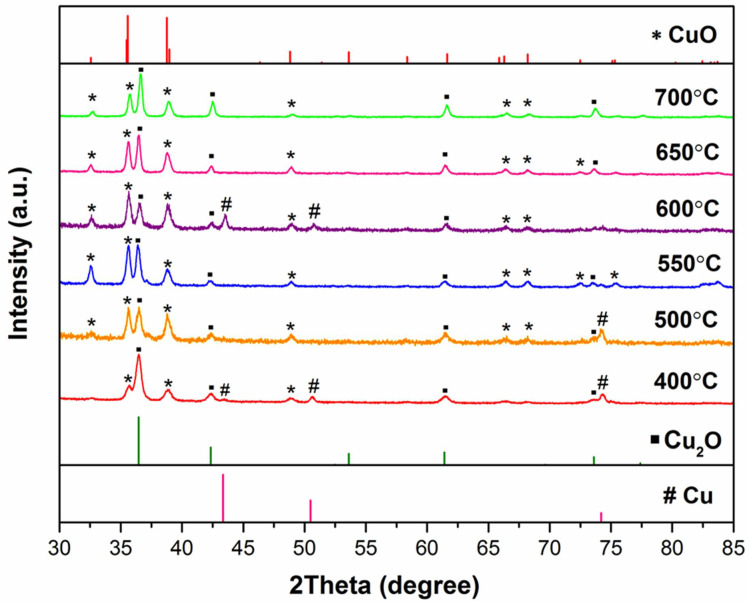
XRD patterns of Cu foils oxidized at different temperatures for 2 h.

The XRD patterns of the samples fabricated at different oxidation temperatures are shown in Figure 2. Two distinct oxide phases, namely cubic Cu_2_O (space group *P*n-3m, JCPDS #05-0667) and monoclinic CuO (space group *C*2/c, JCPDS #45-0937), can be identified in all samples. At the oxidation temperature of 400 °C, the dominant phase is Cu_2_O, with the major peak being Cu_2_O (1 1 1) at 2θ = 36.45°. More CuO is formed as the oxidation temperature increases, which is reflected by the rising intensities of CuO diffraction peaks, especially the major peak of CuO (0 0 2) at 2θ = 35.58°. Some peaks ascribed to face-centered cubic Cu (space group *F*m-3m, JCPDS #04-0836) are also present in some spectra with low intensities. This could be due to the partial exposure of the Cu substrate as a result of non-uniform oxidation process, thus, they are omitted in the quantification of the oxide compositions as shown in Table 2. There is a clear trend that the composition of CuO shows a Gaussian distribution with respect to oxidation temperatures; the CuO/Cu_2_O composition ratio increases to the maximum at 600 °C, and starts to drop beyond this temperature.

**Figure 3:**
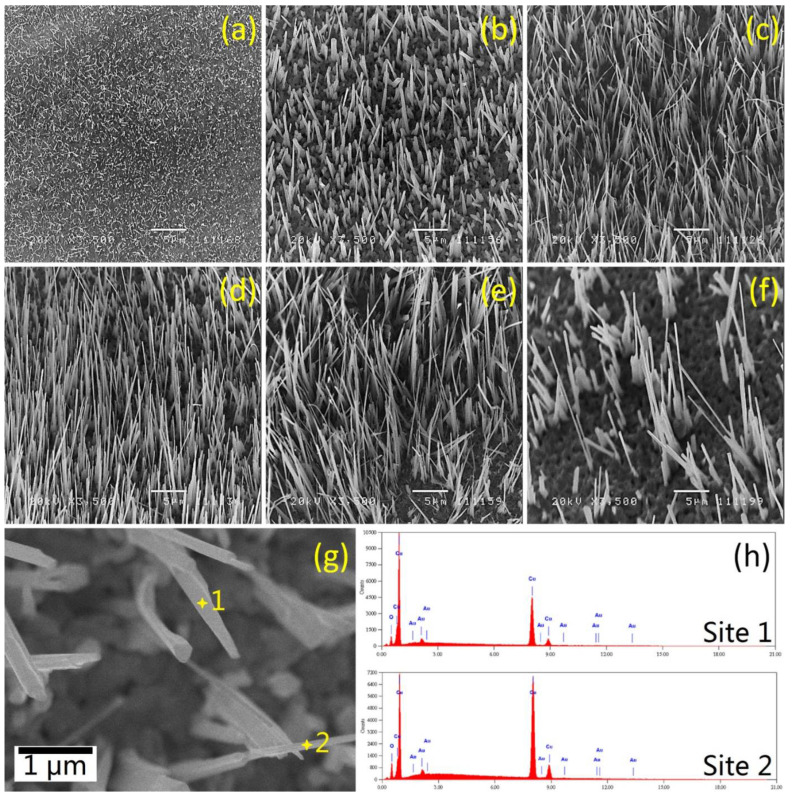
SEM images of (**a**) CuO-400, (**b**) CuO-500, (**c**) CuO-550, (**d**) CuO-600, (**e**) CuO-650 and (**f**) CuO-700; (**g**) magnified SEM image of CuO-600 and (**h**) the corresponding XEDS spectra for the two spot sites labelled in (**g**).

The morphologies of the Cu foils oxidized from 400–700 °C for 2 h were analyzed by SEM under the same magnification of 3500×. As shown in Figure 3, both the density and length of CuO NWs are significantly affected by oxidation temperatures. The density of CuO NWs tends to show a Gaussian distribution in terms of oxidation temperatures. At a low annealing temperature of 400 °C (Figure 3a), the CuO NWs are short, tiny and thin. By increasing the oxidation temperature, both the density and length of CuO NWs increase and reach the maximum at 600 °C. After that, the density and length of CuO NWs start to decrease. At a higher oxidation temperature of 700 °C (Figure 3f), the CuO NWs are sparse and thick. Thus, oxidation of Cu foils at 600 °C (Figure 3d) is found to be optimum for the growth of CuO NWs, which yields thin and straight CuO NWs with the highest density. The above is in good agreement with the XRD results revealed in Figure 2 and Table 2.

A magnified image of CuO-600 is shown in Figure 3g, which shows that the diameter of the as-fabricated CuO NWs is ~200 nm. Two spot sites were randomly picked for XEDS analyses, and the corresponding spectra are displayed in Figure 3h. The elemental compositions (in wt%) of surface species are listed in Table 3. By comparing the experimental results against theoretical values, the elemental compositions of CuO-600 °C are suggested to be CuO, which is aligned with other reported studies [30,31]. Note that Au signals in the XEDS spectra come from the conductive coating during sample preparation.

### 3.2. Electrocatalytic Activities of CuO NWs on Glucose Sensing

**Figure 4:**
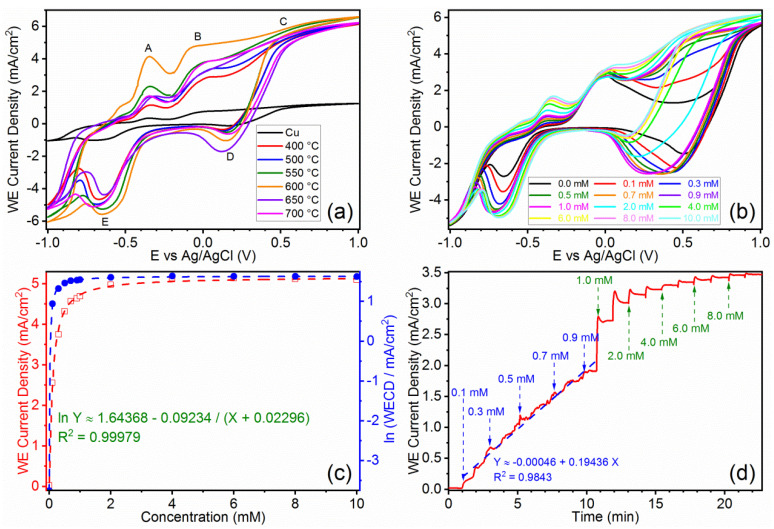
Electrochemistry-based glucose biosensing tests. (**a**) CV curves of samples subjected to different heat treatment temperatures (tested in 10 mM glucose). (**b**,**c**) Sensitivity test of CuO-600, comprising of (**b**) CV curves in various concentrations of glucose and (**c**) the corresponding trend lines. (**d**) CA curve with sequential addition of glucose under constant stirring.

To determine the capability of the as-prepared samples, an initial set of CV measurements were conducted using an electrolyte with 10 mM glucose at pH = 13, and the results are displayed in Figure 4a. For the as-prepared samples, partial exposure of Cu substrate to the electrolyte is unavoidable; three anodic (oxidation) peaks (marked as A–C) and two cathodic (reduction) peaks (marked as D–E) could be identified, as shown in Figure 4a. Peak A represents the oxidation of exposed Cu to Cu (I) species (as given in Equations (1a) and (1b) [32], and Peak B represents the oxidation of the exposed Cu or the as-formed Cu (I) species to Cu (II) species (as given in Equations (1c) and (1e)).

Peak C at 0.5 V is the characteristic signal for glucose detection, as it only appears when glucose is added in the electrolyte, as shown in Figure 4b. The applied voltage of 0.5 V is also the optimum voltage with the highest response sensitivity for glucose, which is aligned with past research [33,34,35,36]. This peak corresponds to the formation of Cu (III) species during the electrochemical process (Equation (1f)), which are responsible for the oxidation of glucose to gluconolactone (Equation (1g)). Abd el Haleem et al. also proposed that Cu (III) species can be detected in strong alkaline solutions, in which the OH^−^ concentration is at least 0.1 M [37]. Thus, the presence of Cu (III) species is considered as an electron mediator that plays an important role in glucose oxidation [38,39,40,41,42,43,44]. On the other hand, as shown in Figure 4b (black curve), peak C is missing due to the absence of glucose in the electrolyte, but peak D is still present. Therefore, peak D should represent the reduction of Cu (II) species to Cu (I) species (Equation (1h)), and peak E represents the reduction of remaining Cu (II) or Cu (I) species formed back to Cu (Equations (1i) and (1j)). By applying a potential of ~0.05 V, thin layers of Cu (II) has been formed on the exposed Cu surface [32], and it should be able to get reduced easily in two steps during the reverse scan.

Equations (1a)–(1j). Electrochemical reactions during cyclic voltammetry:

Peak A: Oxidation of Cu (0) to Cu (I)

(1a) $$\text{Cu}+{\text{OH}}^{-}\to \text{CuOH}+{\text{e}}^{-}$$

(1b) $$2\text{CuOH}⇌{\text{Cu}}_{2}\text{O}+{\text{H}}_{2}\text{O}$$

Peak B: Oxidation of Cu (0)/Cu (I) to Cu (II)

(1c) $$\text{Cu}+2{\text{OH}}^{-}\to \text{Cu}{\left(\text{OH}\right)}_{2}+2{\text{e}}^{-}$$

(1d) $${\text{Cu}}_{2}\text{O}+{\text{H}}_{2}\text{O}+2{\text{OH}}^{-}\to 2\text{Cu}{\left(\text{OH}\right)}_{2}+2{\text{e}}^{-}$$

(1e) $$\text{Cu}{\left(\text{OH}\right)}_{2}⇌\text{CuO}+{\text{H}}_{2}\text{O}$$

Peak C: Formation of Cu (III) species for glucose oxidation

(1f) $$\text{CuO}+{\text{OH}}^{-}\to \text{CuOOH}+{\text{e}}^{-}$$

(1g) $$2\text{CuOOH}+\text{glucose}\to \text{CuO}+\text{gluconolactone}+{\text{H}}_{2}\text{O}$$

Peak D: Reduction of Cu (II) to Cu (I)

(1h) $$2\text{CuO}+{\text{H}}_{2}\text{O}+2{\text{e}}^{-}\to {\text{Cu}}_{2}\text{O}+2{\text{OH}}^{-}$$

Peak E: Reduction of Cu (II)/Cu (I) to Cu (0)

(1i) $$\text{CuO}+{\text{H}}_{2}\text{O}+2{\text{e}}^{-}\to \text{Cu}+2{\text{OH}}^{-}$$

(1j) $${\text{Cu}}_{2}\text{O}+{\text{H}}_{2}\text{O}+2{\text{e}}^{-}\to 2\text{Cu}+2{\text{OH}}^{-}$$

Figure 4c shows the corresponding trend lines of CuO-600. The sensitivity of the sample is given by the plot of WECD against glucose concentration (red profile), which fits perfectly with an exponential function. Sharp increase of WECD is observed at low glucose concentrations (<1 mM) and gradually decelerates above 1 mM, which finally reaches a near-constant value at 4 mM. This value falls right below the designated range of glucose concentrations for impaired fasting glucose (100–125 mg/dL or 5.6–6.9 mM in equivalence) [45,46,47,48]. Plotting the same profile with natural logarithm of WECD against glucose concentrations (blue profile) returns an even lower saturation concentration (~0.7 mM), implying that CuO-600 sample has excellent sensitivity towards glucose intolerance, which is a useful factor for early symptoms of pre diabetes and type 2 diabetes.

CA was used to study the response of CuO-600 to glucose concentrations (as shown in Figure 4d). The test was carried out in an electrolyte containing 0.1 M KOH solution at the applied potential of 0.5 V (vs. Ag/AgCl), and 0.1 mM glucose solutions were successively added under constant stirring. The result shows that at concentrations below 1 mM, the response is almost linearly correlated to glucose concentrations, indicating that the sample could adapt to a quick dynamic change in the environment with a high sensitivity of ~2 mA/cm^2^·mM. Starting from 11 min (1.0 mM glucose), as the amount of glucose is effectively large, and becomes no longer negligible in the resultant electrolyte, a sudden increase in the current density is observed after each subsequent injection, which requires a short period of time to achieve a stable state. Hence, at glucose concentrations above 1 mM, a relatively steady and fast response can be achieved, indicating that the quantitative accuracy of the sample likely starts from a glucose concentration of 1 mM.

## 4. Conclusions

In this work, CuO NWs were successfully grown on the surface of Cu foils with the simple thermal oxidation method. The phase and morphology of the CuO NW can be significantly affected by oxidation temperatures; 600 °C is the optimum value to yield thin and straight CuO NWs with the highest density and oxide phase composition, which facilitates the formation of the Cu (III) species which plays an important role in glucose oxidation. CuO NWs fabricated at 600 °C were found to give the highest sensitivity towards glucose oxidation. While adapting to a quick, dynamic change in the environment, the CuO NWs have a lower detection limit of ~0.1 mM, a low saturation concentration of 0.7 mM, and a lower limit of quantitative accuracy of ~1 mM, which is far below the standard value for impaired fasting glucose, making it an excellent composite biosensor towards early symptom of pre diabetes.

## Figures and Tables

**Table 1:** Comparison of performance for various types of glucose biosensors.

Sample	Type	Sensitivity (mA/cm^2^·mM)	Lower Detection Limit	Reference
CeO_2_ nanorods	EGB	1.65 × 10^−4^	0.1 mM	[22]
ZnO NWs on Au	EGB	0.0195	N. A.	[23]
MnO_2_ decorated graphene nanoribbons	EGB	0.05632	0.05 mM	[24]
ZnO/Pt/chitosan	EGB	0.06214	16.6 μM	[25]
Graphene-based FET	EGB	0.0025	0.1 mM	[26]
CuO/Pt modified Ta_2_O_5_ honeycomb	NEGB	0.16	1 μM	[27]
PEDOT:PSS-CuO-MWCNT	NEGB	0.6632	N. A.	[28]
Au@Cu_2_O	NEGB	0.715	18 μM	[11]
Cu-Cu_2_O decorated graphene foam	NEGB	0.23	16 μM	[29]
ZnFe_2_O_4_ NPs	NEGB	~0.0429	0.1 mM	[20]
CuO NWs	NEGB	~2	0.1 mM	This work

**Table 2:** The composition of oxide species on the surface of Cu foil.

Sample	Cu_2_O	CuO
CuO-400	77.3%	22.7%
CuO-500	49.3%	50.7%
CuO-550	48.1%	51.9%
CuO-600	26.3%	73.7%
CuO-650	40.7%	59.3%
CuO-700	56.9%	43.1%

**Table 3:** The elemental compositions of surface species for CuO-600.

Spot Site	Cu	O	Cu:O
Site 1	75.80 wt%	19.64 wt%	3.859470:1
Site 2	77.39 wt%	19.82 wt%	3.904642:1
Theoretical value for CuO	63.55 wt%	16.00 wt%	3.971875:1
Theoretical value for Cu_2_O	63.55 wt%	8.00 wt%	7.943750:1

## Data Availability

The data presented in this study are available on request from the corresponding author.
